# Expression profiling of Dexamethasone-treated primary chondrocytes identifies targets of glucocorticoid signalling in endochondral bone development

**DOI:** 10.1186/1471-2164-8-205

**Published:** 2007-07-01

**Authors:** Claudine G James, Veronica Ulici, Jan Tuckermann, T Michael Underhill, Frank Beier

**Affiliations:** 1 CIHR Group in Skeletal Development and Remodelling, Department of Physiology and Pharmacology, Schulich School of Medicine and Dentistry, University of Western Ontario, London, Canada; 2 Group of Tissue Specific Hormone Action, Leibniz Institute for Age Research -Fritz Lipmann Institute, Beutenbergstraße 11, D-07745 Jena, Germany; 3 Department of Cellular & Physiological Sciences, University of British Columbia, Vancouver, British Columbia, Canada

## Abstract

**Background:**

Glucocorticoids (GCs) are widely used anti-inflammatory drugs. While useful in clinical practice, patients taking GCs often suffer from skeletal side effects including growth retardation in children and adolescents, and decreased bone quality in adults. On a physiological level, GCs have been implicated in the regulation of chondrogenesis and osteoblast differentiation, as well as maintaining homeostasis in cartilage and bone. We identified the glucocorticoid receptor (GR) as a potential regulator of chondrocyte hypertrophy in a microarray screen of primary limb bud mesenchyme micromass cultures. Some targets of GC regulation in chondrogenesis are known, but the global effects of pharmacological GC doses on chondrocyte gene expression have not been comprehensively evaluated.

**Results:**

This study systematically identifies a spectrum of GC target genes in embryonic growth plate chondrocytes treated with a synthetic GR agonist, dexamethasone (DEX), at 6 and 24 hrs. Conventional analysis of this data set and gene set enrichment analysis (GSEA) was performed. Transcripts associated with metabolism were enriched in the DEX condition along with extracellular matrix genes. In contrast, a subset of growth factors and cytokines were negatively correlated with DEX treatment. Comparing DEX-induced gene expression data to developmental changes in gene expression in micromass cultures revealed an additional layer of complexity in which DEX maintains the expression of certain chondrocyte marker genes while inhibiting factors that promote vascularization and ultimately ossification of the cartilaginous template.

**Conclusion:**

Together, these results provide insight into the mechanisms and major molecular classes functioning downstream of DEX in primary chondrocytes. In addition, comparison of our data with microarray studies of DEX treatment in other cell types demonstrated that the majority of DEX effects are tissue-specific. This study provides novel insights into the effects of pharmacological GC on chondrocyte gene transcription and establishes the foundation for subsequent functional studies.

## Background

Cartilage provides a scaffold for the deposition of osteoblast precursors and ultimately the development of long bones. This process, termed endochondral ossification, describes a coordinated developmental series that involves commitment of mesenchymal precursor cells to the chondrogenic lineage and subsequent alternating phases of proliferation and differentiation, which culminate in the replacement of the cartilage by bone tissue [1-4]. In the first phase of this process, multipotent mesenchymal progenitors condense and initiate expression of the pro-chondrogenic Sox family members 9, 5 and 6 [5,6]. A subset of cells at the center of these aggregates differentiates into chondrocytes. Newly formed chondrocytes secrete an extracellular matrix rich in type II collagen (*Col2a1*), proliferate and ultimately terminally differentiate into hypertrophic chondrocytes [7]. Chondrocyte hypertrophy precedes the end of the chondrocyte life cycle by apoptosis and is accompanied by vascularization of the hypertrophic template and mineralization of the cartilaginous extracellular matrix [8-12]. Concomitantly, osteoclasts degrade the calcified cartilage extracellular matrix, making way for the invasion and deposition of an osteoprogenitor population that form the primary ossification center [13].

These events take place in a region called the growth plate that illustrates the organization of different phases of cartilage development into distinct zones. The resting zone delineates newly differentiated chondrocytes with low mitotic activity and the cellular reserve for subsequent stages of chondrocyte differentiation. Proliferative zone chondrocytes exhibit higher mitotic activity resulting in distinct columns containing cells reminiscent of stacked coins. The hypertrophic zone demarcates terminally differentiated chondrocytes which are identified by high cytoplasm to nuclear ratio and the expression of type X collagen (Col10a1) [14-16]. Terminally differentiated chondrocytes are fated for programmed cell death after which primary ossification occurs by way of vascularization of the remaining cartilaginous matrix and the deposition of osteoprogenitor cells [17-19].

Glucocorticoids (GC) are among various endocrine molecules including growth hormone (GH) and thyroid hormone (TH) known to regulate linear growth [20-23]. Regulation of linear growth follows the paradigm in which steroid hormones affect target tissue through both local and systemic mechanisms [24-27]. Indirect effects occur through modulation of other endocrine systems such as the GH/IGF-I axis. Generally, GC decrease IGF-I, GH receptor and IGF receptor 1 expression and also abrogate the release of GH from the pituitary [20,28,29]. Direct regulation of growth occurs through GC receptor (GR)-mediated gene transcription in chondrocytes [24,30,31].

GC functions are primarily mediated by the glucocorticoid receptor (GR) that is encoded by the *Nr3c1 *gene. The GR is ubiquitously expressed in mammalian tissues, including the growth plate, and is essential for life [31-36]. Many studies have examined GC regulation of the skeleton and have led to various theories on potential modes of GC function in cartilage [37-40]. The specific function of the receptor in terms of its transcriptional regulation in cartilage, however, remains enigmatic.

While endogenous GCs have been shown to promote the differentiation of both chondrocytes and osteoblasts, exogenous GCs in pharmacological doses which are also widely used in clinical practice to treat inflammatory disorders [41-46]. Their have different effects. Indeed, their utility in treating various diseases is, however, limited by numerous side effects such as growth failure and decreased bone quality [47]. GC-target genes including C-type natriuretic peptide and VEGF have been identified in chondrocytes [28,48,49]; however, the cartilage-specific transcriptional consequences of high-GC-doses in the growth plate have not been studied comprehensively.

Work in our laboratory identified GR amongst factors that were up-regulated during chondrocyte maturation [50] Thus, to comprehensively understand the transcriptional effects of pharmacological GC doses in growth plate, we completed a genomic screen of gene expression changes in chondrocytes derived from E15.5 day old mouse embryos. Primary monolayer chondrocytes were treated with a synthetic GC, dexamethasone (DEX), and RNA was isolated for microarray analysis. We complemented traditional microarray analysis methods with the gene set enrichment algorithm to correlate the behaviour of specific molecular classes with DEX treatment [51,52].

## Results and Discussion

### Microarray screen of dexamethasone-treated primary chondrocyte monolayers

**Figure 1:**
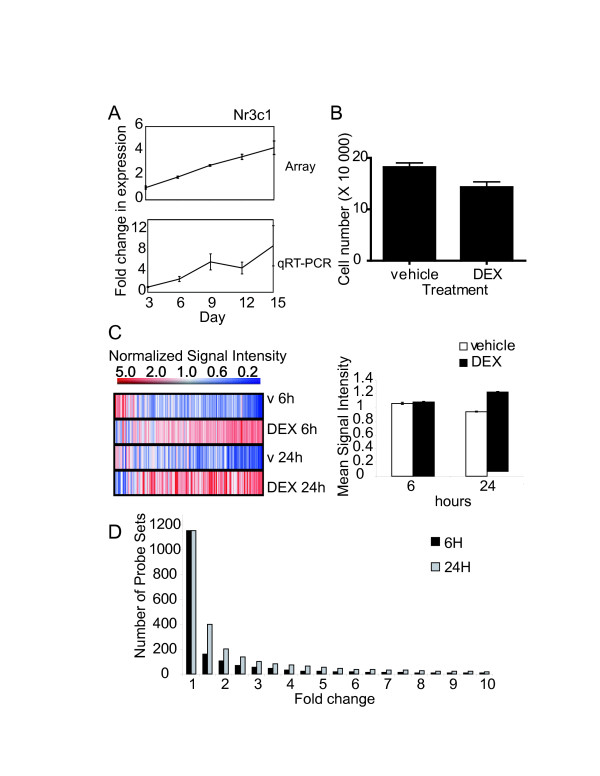
**Gene expression changes in DEX-treated primary chondrocytes**. Microarray and quantitative RT-PCR expression profiles of the Glucocorticoid receptor (*Nr3c1*) in primary mesenchymal micromass cultures (A). Primary chondrocytes are plated in high density monolayers and treated with DEX or vehicle for 24 hrs and counted with a hemocytometer (B). Ordered list of global microarray data set derived from the hybridization of RNA isolated from primary chondrocytes treated with 10^-7 ^M DEX and the vehicle (v) control (C, left panel). One-Way ANOVA testing for significantly expressed probe sets between DEX-treated samples and the vehicle control resulted in a list of 1158 transcripts. Mean normalized signal intensities for all 1158 probe sets are shown (C, right panel). Fold change filtering of these transcripts reveal that the majority of probe sets vary in the range of 1 to 2-fold (D).

We identified the GR as a candidate for the regulation of chondrocyte hypertrophy in a previous expression profiling screen using primary micromass cultures [50]. The *Nr3c1 *probe set which encodes the GR was up-regulated 4-fold from day 3 to day 15 of micromass culture (Figure 1A, top panel). Confirmation of the GR expression profile with qRT-PCR showed an approximately 8-fold increase over the same time course (Figure 1A, bottom panel). Studies in our laboratory and others have implicated GCs in chondrocyte differentiation and growth plate function [25,26,47,48,53,54]. In addition, our cell counting experiments revealed that DEX consistently decreases cell numbers after 24 hrs (Figure 1B), in agreement with other studies that show increased apoptosis [38,55] and reduced proliferation [56] in response to GCs. We therefore aimed at extending this analysis to examine pharmacological effects of GCs on growth plate chondrocytes by systematically identifying downstream effector genes of DEX. Primary chondrocytes derived from the long bones of 15.5 day old embryonic mice were treated with DEX or the vehicle control, and total RNA was isolated after 6 and 24 hrs of culture, respectively.

Gene expression was evaluated using Affymetrix MOE 430 2.0 mouse genome chips using three independent cell isolations. We first analyzed gene expression using conventional analysis functions in GeneSpring GX*. After pre-processing the data set using the GC-RMA algorithm and eliminating probe sets showing expression levels close to background, 22 091 probe sets remained, reducing the data set by 48% (Table 1). Significance testing with one-Way ANOVA analysis identified probe sets differentially expressed between DEX and vehicle-treated cultures over the entire time course (Figure 1C, left panel). The resulting list contained 1158 probe sets, which is 2% of the data set's original size. Approximately 70% of significantly changed probe sets exhibited upregulation in response to DEX treatment. This data set was further subdivided by using 1.5-, 5- and 10- fold change filters which generated lists of 162, 21 and 7 probe sets for the 6 hr time point and 399, 53 and 19 probe sets for the 24 hr time point, respectively (Table 1). Examination of the overall differences between the mean normalized signal intensities associated with each condition showed minimal changes in gene expression (Figure 1C, right panel), indicating that GC treatment affects the expression of only a small subset of all expressed genes in this system. A distribution of fold differences between 6 and 24 hrs showed that the majority of gene expression changes did not exceed 2-fold (Figure 1D). In each case, both time points exhibited the same overall trends in gene expression, but, as expected, the 24 hr time point consistently showed a higher proportion of probe sets altered by DEX treatment.

**Table 1:** Microarray analysis of DEX-treated primary chondrocyte monolayers.

Specifications	Probe sets at 6 hrs	Probe sets at 24 hrs
**Total number of probe sets**	**45101**	**45101**
Significantly expressed	22091	22091
**Differentially expressed**	**1158**	**1158**
1.5-fold changed	162	399
**5-fold changed**	**21**	**53**
10-fold changed	7	33
**1.5-fold up-regulated**	**141**	**342**
5-fold up-regulated	20	50
**10-fold up-regulated**	**7**	**19**
1.5-fold down-regulated	21	57
**5-fold down-regulated**	**1**	**3**
10-fold down-regulated	0	0

### Probe set validation

**Figure 2:**
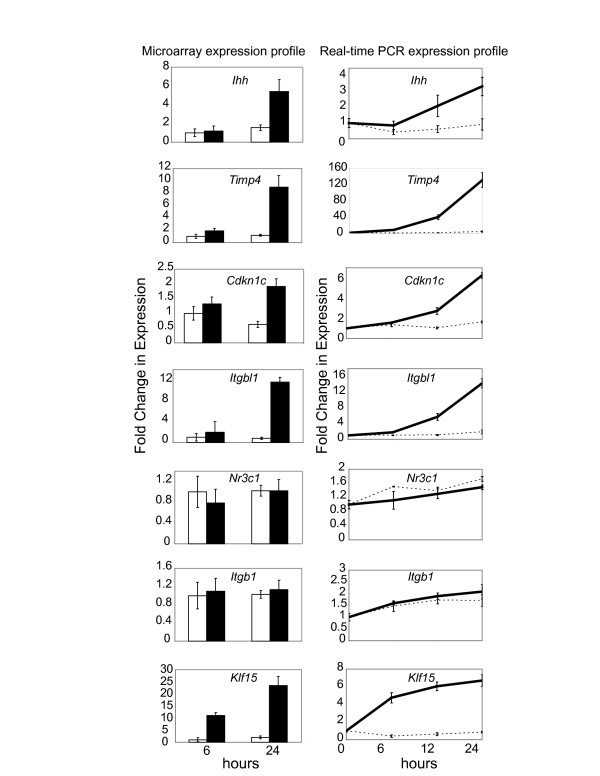
**Identification of significantly expressed probe sets and subsequent validation with real-time RT-PCR**. Expression profiles for selected transcripts in vehicle- or DEX-treated chondrocytes are confirmed with real-time RT-PCR at 0, 6, 12 and 24 hr time points. Indian hedgehog (*Ihh*), tissue inhibitor of matrix metalloproteinase 4 (*Timp4*), cyclin-dependent kinase inhibitor 1C (*Cdkn1c*, p57), integrin beta like 1 protein (*Itgbl1*), glucocorticoid receptor (*Nr3c1*), integrin beta 1 (*Itgb1*) and kruppel-like factor 15 (*Klf15*) microarray data are shown on the left at the 6 and 24 hr time points and corresponding real-time expression values are shown on the right. P-values less than 0.01 are deemed significant. Specifically, *Ihh*, *Timp4*, *Itgbl1 *and *Klf15 *exhibit significant differences between the 6 and 24 hr time point and between treatments. Dotted lines indicate the control and solid lines denote DEX treatment.

To confirm the accuracy of the microarrays in identifying biologically significant differences, we selected a variety of expressed transcripts for qRT-PCR analysis (Figure 2A). Transcripts that either belonged to a functional class implicated in cartilage development or exhibited marked changes with DEX treatment were chosen. Markers exhibiting marginal changes in gene expression were also selected for control purposes. Specifically, we evaluated the expression patterns of Indian hedgehog (*Ihh*), Tissue inhibitor of matrix metalloproteinase 4 (*Timp4*), Cyclin-dependent kinase inhibitor 1C (*Cdkn1c*), which contains a GC response element in its promoter [57], Integrin beta like 1 protein (*Itgbl1*), GC receptor (*Nr3c1*), Integrin beta 1 (*Itgb1*) and Kruppel-like factor 15 (*Klf15*) over 0, 6, 12, and 24 hrs of culture with or without DEX treatment. Transcripts for *Klf15 *were up-regulated from 0 to 6 hrs while *Ihh*, *Timp4*, *Cdkn1c *and *Itgbl1 *all increased after the 6 hr time point. *Nr3c1*, which encodes the GR, was not affected by DEX-treatment at both 6 and 24 hrs, but does contain a putative GRE [58]. Transcripts such as *Itgb1 *that exhibited less than 1.5-fold change in our arrays were also confirmed with qRT-PCR, providing further evidence that the microarray data represented authentic gene expression data. Interestingly, the fold change difference varied according to the experimental method. In cases such as *Timp4 *and to a lesser extent *Cdkn1c*, qtPCR data showed higher fold change increases with the DEX treatment than in microarrays. In contrast, the expression pattern for *Klf15 *exhibits a higher fold-change difference in the microarrays compared to the control. While data normalization using the RMA algorithm provides excellent estimates of reliable signal intensities, other methods such as the M.A.S. 5.0 algorithm are known to outperform RMA in its ability to accurately estimate fold change differences in transcript levels [59].

### GSEA to identify the effects of dexamethasone on gene expression in chondrocytes

Traditional microarray analysis methods are useful for the identification of probe sets exhibiting transcriptional responses to DEX-treatment, but are limited in certain capacities. Alternate statistical methods such as ANOVA testing produced transcript lists that, while effectively reducing the dimensionality or sample size of the data set, increased the rate of false negative data thus hampering our ability to generate meaningful hypotheses from the data (Figure 1). Also, the overall effect of DEX treatment on gene expression was modest, which may have reduced the significance of biologically relevant genes because their signal intensities were close to background levels. Accordingly, we did not have a clear concept of the central pathways and biological categories affected by DEX treatment. Similarly, Gene Ontology annotations were not sufficiently robust to detect differences in the representation of specific molecular categories (data not shown). We therefore implemented GSEA [52], an algorithm that is designed to effectively evaluate the effect of a specific experimental condition on known biological pathways and functional categories. These analyses show whether a given treatment (e.g. DEX stimulation) results in enrichment of genes sets involved in the regulation of a specific phenotype (see materials and methods for details).

**Figure 3:**
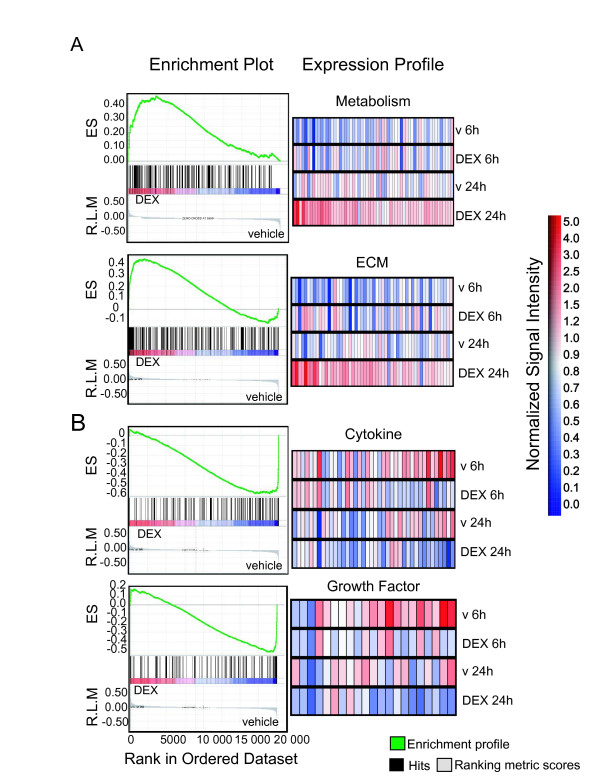
**Enrichment plots for statistically significant gene sets identified by GSEA**. User-defined gene sets enriched with the DEX or vehicle conditions are depicted. Black bars illustrate the position of probe sets belonging to metabolic, extracellular matrix (A), cytokine and growth factor (B) gene sets in the context of all probes on the DEX array. The running enrichment score (RES) plotted as a function of the position within the ranked list of array probes is shown in green. The ranked list metric shown in gray illustrates the correlation between the signal to noise values of all individually ranked genes according and the class labels (experimental conditions). Metabolic and ECM genes are overrepresented in the left side of the enrichment plot indicating correlation to differential expression in DEX-treated chondrocytes. In contrast, cytokines and growth factor genes are enriched in the right side of the plots and correspond to the vehicle control. Significantly enriched data sets are defined according to GSEA default settings i.e., a p < 0.001 and a false discovery rate (FDR) < 0.25. Individual expression profiles for probe sets contributing to the normalized enrichment score are shown in the right panel. R.L.M = ranked list metric, E.S. = enrichment score.

**Figure 4:**
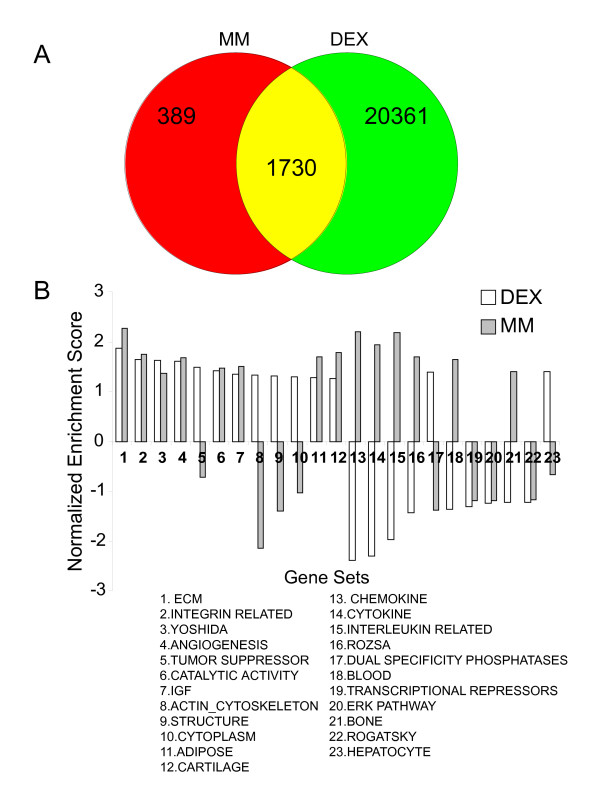
**Comparison of DEX-treated primary chondrocytes to a time course of chondrocyte differentiation in micromass culture**. The Venn diagram depicts probe sets that are common between the list of 2119 probe sets differentially expressed between days 3 and 15 of micromass culture and the list of 22 091 significantly expressed probe sets in primary chondrocyte monolayer cultures (A). The matrix of 77 user-defined gene sets are used to interrogate microarray data from days 15 and day 3 of micromass culture. Normalized enrichment scores (NES) generated from this analysis are then compared to NES scores derived from the DEX study to evaluate similarities in the regulation of different groups of genes in chondrocytes (B). Positive enrichment scores (ES) indicate gene sets that are enriched and up-regulated in DEX-treated chondrocytes or d15 of micromass culture. Negative ES indicate gene set enrichment and down-regulation in the DEX-treatment or up-regulation in the day 3 samples of the micromass (MM) culture data set.

We created a gene set consisting of 77 gene lists representing different tissue types, functional categories and pathways derived from other microarray studies in the literature (Table 2). We drew conclusions from the top gene sets that had a false discovery rate (FDR) less than 25% and a p-value less than 0.001, both of which are acceptable cut-offs for the identification of biologically relevant probe sets. This cut-off, although relatively high, was optimized to reduce the occurrence of false negative data in data sets interrogating a small number of gene sets. Additionally, the FDR compensates for the inherent lack of coherence microarray data sets exhibit between gene expression and specific experimental conditions [52]. Enriched gene sets were identified in both DEX and vehicle data (Table 3). Specifically, the highest statistical confidence and correlation with the DEX phenotype was assigned to metabolism and extracellular matrix, which contained 196 and 228 genes, respectively (Figure 3, left panels, Table 4 and 5). In each case, the expression of genes positively correlated with the DEX phenotype at the 24 hr time point exceeded the number of genes at the 6 hr time point (Figure 3, right panels). Metabolic genes included aldehyde and alcohol dehydrogenases (Table 4), among others, and were identified in accordance with previously documented roles for GC in various metabolic processes and tissues [60,61]. Closer examination of the genes contributing to the enrichment scores for the ECM gene set revealed that Dentin matrix protein 1 (*Dmp1*) was the top ranking gene (Table 5). DMP1 belongs to the SIBLING family of matrix molecules and has been linked to chondrocyte differentiation. *Dmp1 *knockout mice display disordered postnatal chondrogenesis, among other skeletal abnormalities [62]. Interestingly, integrin binding sialoprotein (*Ibsp*) [63-66]), another SIBLING family member, and osteocalcin (*Bglap2*) both contain putative GRE sequences, but did not contribute to the enrichment score for this category [63,66]. They did, however, belong to the core group of genes that were enriched when a micromass culture gene set was used to interrogate the DEX data (Figure 4).

**Table 2:** Gene sets used in GSEA.

Category name	Number of genes	Category name	Number of genes
Adipose	70	Nucleus_3	510
Apoptosis	39	Fkbp	33
Bone	116	3vs15_1.5x_1	497
Cartilage	28	3vs15_1.5x_2	497
Catalytic	245	3vs15_1.5x_3	497
Chaperone	81	3vs15_1.5x_4	497
Chemokine	31	3vs15_1.5x_5	76
Chromatin/Hdacs	24	Igf	48
Cyclin	225	Cart_2	299
Cytokine	127	Cart_3	352
1_Dnabind	500	Liver_1	260
2_Dnabind	448	Liver_2	260
Ecm	228	Blood	111
Electron_Transp	40	Protease_1	269
Gf Receptor	327	Protease_2	269
Gluconeogen	31	Phosphatase	473
Growth Factor	106	Dusp	20
Gtpase Activator	46	Kinase_1	499
Gtpase Activ	73	Kinase_2	499
Heparin Bind	37	Kinase_3	227
Hormone	75	Integrin_Rel	173
Muscle	198	Brain_Rel	379
Neg_Apoptosis	50	Hepatocyte	19
Oncogene	154	Obl_Oclast	16
Pos_Apoptosis	79	Interleukinrelated	175
Related_Apoptosis	311	Rgs_Related	44
Structure	151	Caspase_Related	47
Sugar_Bind	104	Creb_Atf3	32
Tf_Activ	56	Nuclear Receptor	138
Tf_Repress	55	Nuc_Hormone_Receptor	55
Tgfb	45	Mapkrelated	267
Tnf_Receptor	69	Membrane	260
Tumor Suppressor	48	Metabolism	196
Wnt	53	Nucleus_1	494
Actin_Cytoskel	38	Nucleus_2	494
Angiogen	57	Pzhorton.Farnum	413
Bmprelated	62	Hzhorton.Farnum	407
Cytoplasm	411		
Erk_Related	40		
Fgf_Related	64		

**Table 3:** GSEA of DEX-treated primary chondrocytes.

Gene set name	Size	ES	NES	NOM p-val	FDR q-val
Metabolism	196	0.471	1.935	<0.001	0.016
Extracellualr Matrix	228	0.451	1.878	<0.001	0.016
Fkbp	33	0.559	1.696	0.011	0.054
Integrin_Related	173	0.407	1.643	<0.001	0.001
Angiogenesis	57	0.479	1.610	0.012	0.065
Kinase_1	499	0.343	1.549	<0.001	0.092
Tumor Suppressor	48	0.457	1.492	0.037	0.126
Catalytic	245	0.337	1.420	0.008	0.172
Hepatocyte	19	0.529	1.406	0.104	0.161
D3 Vs D15_2	497	0.304	1.368	0.004	0.194
Igf	48	0.412	1.348	0.093	0.208
Cyclin	224	0.322	1.344	0.028	0.199
Actin_Cytoskel	38	0.426	1.325	0.124	0.213
Structure	151	0.332	1.312	0.053	0.219
Cytoplasm	411	0.292	1.300	0.023	0.224
Adipose	70	0.368	1.285	0.116	0.232
Gtpase Activity	73	0.363	1.280	0.113	0.230
Cartilage	28	0.432	1.262	0.169	0.246
Chemokine	31	-0.779	-2.40	<0.001	0
Cytokine	127	-0.579	-2.31	<0.001	0
Growth Factor	106	-0.517	-2.01	<0.001	7.698E-04
Interleukinrelated	175	-0.469	-1.98	<0.001	9.475E-04
Bone	16	-0.577	-1.51	0.051	8.945E-02
Creb_Atf3	30	-0.469	-1.43	0.065	1.300E-01
Dusp	20	-0.508	-1.40	0.102	1.418E-01
Blood	111	-0.351	-1.37	0.037	1.425E-01
3vs15_1.5x_3	496	-0.288	-1.35	0.002	1.518E-01
Protease_2	268	-0.306	-1.35	0.015	1.411E-01
Nuc_Hormone_Receptor	55	-0.381	-1.32	0.086	1.570E-01
Tf_Repress	55	-0.380	-1.32	0.091	1.498E-01
3vs15_1.5x_4	497	-0.272	-1.28	0.011	1.817E-01
Erk_Related	40	-0.385	-1.25	0.157	2.169E-01

ES, enrichment score

NES, normalized enrichment score

FDR q-val, false discovery rate and multiple testing corrections (q-value)

NOM p-val; the uncorrected p-value

**Table 4:** Metabolic transcripts enriched in DEX-treated chondrocytes. I.

HUGO symbol	Rank	RMS*	RES**	HUGO symbol	Rank	RMS*	RES**
Aldh1a1	26	0.417	0.053	Slc27a4	1616	0.058	0.426
Eya2	40	0.355	0.099	Ltbp2	1721	0.056	0.428
Vcl	106	0.228	0.125	Hsd17b1	1783	0.055	0.432
Adhfe1	116	0.222	0.154	P4ha2	1783	0.055	0.432
Ids	123	0.212	0.181	Mut	1850	0.053	0.443
Cbr3	133	0.204	0.207	Pde3a	2195	0.048	0.432
Aldh6a1	202	0.165	0.225	Sulf2	2200	0.048	0.438
Bcat2	224	0.157	0.245	Prep	2316	0.046	0.438
Pmm1	278	0.145	0.261	Plod3	2387	0.045	0.441
Pcx	553	0.105	0.261	1110013G13RIK	2510	0.043	0.440
Fthfd	554	0.105	0.275	Pld1	2669	0.041	0.437
Atp1a1	560	0.104	0.288	Au041707	2721	0.040	0.440
Gstm1	619	0.099	0.298	Decr1	2837	0.039	0.439
Gstm2	742	0.088	0.303	Gstm5	2872	0.038	0.443
1700061G19RIK	787	0.086	0.312	Bckdha	2932	0.038	0.445
Slc38a4	833	0.084	0.321	Atp11a	2951	0.038	0.449
Pyp	847	0.083	0.331	Gstp1	2967	0.037	0.453
Aacs	901	0.080	0.339	Dhrs7	3014	0.037	0.455
Plod1	934	0.079	0.348	Cbr2	3147	0.035	0.453
Acas2	983	0.077	0.355	Echdc3	3152	0.035	0.458
Auh	1068	0.074	0.361	Acy3	3254	0.035	0.457
Gcat	1109	0.072	0.368	Dhrs1	3483	0.032	0.450
Dhrs8	1184	0.070	0.373	Itgb1	3527	0.032	0.452
Egln3	1232	0.068	0.380	4933406E20RIK	3553	0.031	0.454
Mthfs	1268	0.067	0.387	Plod2	3574	0.031	0.458
Mvk	1298	0.066	0.394	Pmm2	3582	0.031	
Aup1	1325	0.065	0.401	Ugp2	3583	0.031	
Spr	1456	0.062	0.403	Gnpat	3633	0.031	
Sc5dl	1462	0.062	0.411	1110003P22RIK	3636	0.031	
1300018J18RIK	1516	0.061	0.416	Dbt	3710	0.030	
Agpat3	1524	0.061	0.423				

Rank = position of genes in the context of the ranked list of array genes

RMS = the ranked metric score

RES = the running enrichment score

**Table 5:** ECM-related transcripts enriched in DEX-treated chondrocytes.

HUGO symbol	Rank	RMS	RES	HUGO symbol	Rank	RMS*	RES**
Dmp1	18	0.470	0.036	Matn4	882	0.081	0.420
Omd	27	0.409	0.068	Lama3	886	0.081	0.427
Itga5	38	0.358	0.095	Nyx	992	0.077	0.427
Adamts1	57	0.305	0.118	Lamb2	1082	0.073	0.429
Timp4	61	0.296	0.141	Bsg	1100	0.072	0.433
Col4a1	86	0.268	0.161	Fbn2	1242	0.068	0.432
Col4a2	98	0.247	0.180	Ntn4	1245	0.068	0.437
Adam12	112	0.225	0.197	5730577E14RIK	1381	0.064	0.435
Prelp	139	0.200	0.211	Col6a2	1405	0.064	0.439
Postn	142	0.195	0.227	Ntn3	1415	0.063	0.443
Chad	176	0.174	0.239	Tgfb2	1531	0.060	0.442
Mgp	195	0.168	0.251	Mia1	1575	0.059	0.445
Col1a1	232	0.154	0.261	Mmp14	1803	0.054	0.438
Mfap5	233	0.153	0.273	Col15a1	1845	0.053	0.440
Col10a1	266	0.146	0.283	Ctgf	1882	0.052	0.442
Smoc2	279	0.145	0.294	Col6a1	1942	0.052	0.443
Aspn	294	0.141	0.304	Gpld1	1946	0.051	0.447
Col4a5	367	0.128	0.310	Emid2	2043	0.050	0.446
Adamts15	385	0.126	0.319	Col7a1	2047	0.050	0.450
Tgfb1	394	0.125	0.329	Adam10	2107	0.049	0.451
Sparcl1	440	0.119	0.336	Col9a2	605	0.100	0.370
Adam17	483	0.112	0.343	Matn3	610	0.099	0.377
Lama5	508	0.110	0.350	Col11a2	636	0.097	0.384
Lamc1	517	0.109	0.358	Hapln1	650	0.096	0.391
Spock2	581	0.102	0.363	Lama2	685	0.092	0.396
Lama1	688	0.092	0.403	Gpc3	796	0.086	0.412
Ltbp4	704	0.091	0.410	Lama4	827	0.084	0.417

*RMS = the ranked metric score

**RES = the running enrichment score

Osteomodulin, an additional matrix molecule shown to be structurally similar to IBSP [67], ranked second in the list of enriched ECM genes. Additional ECM molecules expressed in terminally differentiated chondrocytes such as collagen 10 (*Col10a1*) and osteonectin (*Spock1*) were identified, suggesting that this molecular classification is important for transmitting GC signaling in the growth plate.

Interestingly, the normalized enrichment scores for factors down-regulated by DEX treatment were higher than those positively correlated with DEX, but contained fewer probe sets contributing to the scores. Gene sets composed of 127 and 106 genes associated with cytokine and growth factor activity, respectively, were negatively correlated with DEX treatment (Figure 4, Table 6, 7). In other studies, cytokines such as Il-8 and GROα were found to promote the hypertrophy of osteoarthritic cartilage, and excess interleukins 1β(IL-1β), interleukin 6 (IL-6) and Tumor Necrosis Factor alpha (TNF-α) cause growth failure in children [68-70]. Our studies identified three members of the GP-130 family of cytokines, namely interleukins -11,-6 (*Il11, Il6*) and leukemia inhibitory factor (*Lif*), as part of the core enrichment group for cytokines (Table 6). Transgenic mice overexpressing Il-6 exhibit growth retardation, and LIF is thought to regulate the rate at which terminally differentiated cartilage is calcified and vascularized [71,72].

**Table 6:** Cytokine transcripts enriched in vehicle-treated chondrocytes. I.

HUGO gene symbol	Rank	RMS	RES
Cklfsf2b	16971	-0.0424	-0.574
Il7	16981	-0.0425	-0.569
Il1f9	17007	-0.0427	-0.566
Grn	17130	-0.0439	-0.567
Il1f6	17153	-0.0442	-0.563
Ifna2	17418	-0.0468	-0.571
Tslp	17503	-0.0477	-0.570
Il17	17568	-0.0483	-0.568
A730028g07rik	17606	-0.0486	-0.564
Cxcl11	17634	-0.0490	-0.560
Ctf1	17857	-0.0519	-0.565
Lta	17864	-0.0519	-0.559
Il1a	18018	-0.0539	-0.561
Ccl20	18038	-0.0542	-0.556
Ccl17	18334	-0.0584	-0.564
Ccl12	18384	-0.0592	-0.560
Cklf	18618	-0.0639	-0.564
Ifna11	18855	-0.0688	-0.568
Cklfsf6	18874	-0.0693	-0.561
Il15	18955	-0.0719	-0.557
Ltb	19146	-0.0779	-0.558
Ccl3	19220	-0.0814	-0.552
Tnfsf9	19228	-0.0816	-0.543
Cx3cl1	19523	-0.0975	-0.547
Gdf15	19660	-0.1100	-0.541
Bmp5	19775	-0.1238	-0.533
Cxcl14	19798	-0.1289	-0.519
Cxcl1	19928	-0.1698	-0.507
Cxcl10	19951	-0.1807	-0.487
Ccl7	19956	-0.1849	-0.466
Gdf5	19973	-0.2066	-0.444
Cxcl12	19978	-0.2104	-0.420
Areg	19983	-0.2189	-0.395
Cxcl2	19996	-0.2421	-0.369
Ppbp	20014	-0.2944	-0.336
Lif	20024	-0.3296	-0.299
Ccl2	20030	-0.3589	-0.258
Il11	20035	-0.4036	-0.213
Cxcl5	20039	-0.5406	-0.152
Tnfsf11	20041	-0.5835	-0.085
Il6	20043	-0.7529	

Rank = position of genes in the context of the ranked list of array genes

RMS = the ranked metric score

RES = the running enrichment score

**Table 7:** Growth factor transcripts vehicle in DEX-treated chondrocytes.

HUGO gene symbol	Rank	RMS	RES
Fgf21	18968	-0.073	-0.508
Nrg3	19132	-0.077	-0.506
Fgf5	19190	-0.080	-0.499
Ereg	19507	-0.096	-0.502
Fgf7	19581	-0.102	-0.493
Gdf15	19660	-0.110	-0.483
Igf1	19679	-0.111	-0.469
Bmp5	19775	-0.124	-0.458
Nov	19848	-0.144	-0.443
Vegf	19877	-0.150	-0.425
Ptn	19885	-0.153	-0.406
Cxcl1	19928	-0.170	-0.386
Bdnf	19939	-0.176	-0.364
Inhba	19971	-0.204	-0.340
Gdf5	19973	-0.207	-0.313
Cxcl12	19978	-0.210	-0.287
Areg	19983	-0.219	-0.259
Hbegf	20006	-0.264	-0.226
Ngfb	20013	-0.287	-0.189
Lif	20024	-0.330	-0.148
Il11	20035	-0.404	-0.096
Il6	20043	-0.753	0.000

Rank = position of genes in the context of the ranked list of array genes

RMS = the ranked metric score

RES = the running enrichment score

This group also contained the gene encoding Tumor necrosis factor (ligand) superfamily, member 11 (*Tnfsf11*, RANKL), which has been localized to mature chondrocytes and is thought to promote degradation of the calcified cartilage ECM and ultimately endochondral ossification through activation of osteoclasts [73-75]. It is important to note that several independent gene sets connected to inflammation such as cytokines, chemokines and interleukins exhibit some overlap and showed similar enrichment patterns, which provides additional confirmation that DEX is indeed downregulating inflammatory molecules in chondrocytes. GC have been previously reported to down-regulate the expression of VEGF, one of the central growth factors involved in vascularization of calcified cartilage matrix [49], in agreement with our data (Table 7). Since some of these factors, such as RANKL, VEGF and LIF, promote normal tissue remodeling processes during endochondral ossification, our data suggest that DEX prevents the replacement of hypertrophic cartilage by bone. GC have been shown to delay chondrocyte maturation while retaining their capacity to re-engage in their developmental program [21]. This could account for upregulation of genes typically associated with the chondrocyte phenotype, such as ECM genes and the coordinated downregulation of factors that promote the transition from cartilage into bone.

### Identification of cartilage-specific dexamethasone-effects

Identification of cartilage-specific gene sets affected by DEX treatment provided further insight into the complex nature of GC functions in cartilage. We knew from other studies that DEX effects on chondrogenic differentiation are dependent on cell source, experimental system and DEX concentration [40,42,76-78]. We aimed to systematically characterize the effects of DEX on growth plate chondrocytes. To ensure that our DEX data set was expressing bona fide cartilage markers, we compared the DEX data to our previously generated micromass culture data set [50]. We compared all expressed probe sets in the DEX array to probe sets exhibiting a minimum 1.5-fold change in expression between days 3 and 15 of micromass cultures that encompass the various stages of the chondrocyte life cycle. Day 3 of micromass culture likely coincides with the onset of the cartilage developmental program and early chondrogenesis. After 15 days of culture, the cell population is comprised primarily of terminally differentiated chondrocytes and thus corresponds mostly to the hypertrophic zone of the growth plate [50,79], although small numbers of other cells are present at all stages. Out of the 2119 probe sets displaying at least 1.5-fold changes in expression in the micromass culture data set (a probe set list generated from the pair-wise comparison of day 3 versus day 15 of micromass culture), 1730 were also expressed in the DEX array. This shows that our primary chondrocyte monolayers do exhibit prototypical chondrocyte gene expression patterns in both the presence and absence of DEX treatment.

To complete more robust classification of the data in which we could correlate chondrocyte gene expression to the DEX phenotype, we created a gene set from this list of 2119 probe sets (Table 8, 9). The micromass derived gene list was enriched in this study; however, the list was found to correlate both positively and negatively with different aspects of the DEX phenotype. We therefore proceeded to evaluate both the micromass (MM) data set and the DEX data set using GSEA analysis and the previously created gene sets. If both the micromass time course and the DEX data sets show the same enrichment pattern, we would have evidence to suggest that pharmacological DEX doses promote chondrocyte differentiation. Normalized enrichment scores for gene sets common to both culture methods were therefore compared to identify differences and similarities between DEX-treated chondrocytes and the chondrocyte phenotype (Figure 4B).

**Table 8:** Micromass culture-derived gene sets are enriched in DEX-treated primary chondrocytes (d3 vs d15_2). I.

HUGO gene symbol	Rank	RMS	RES
Itgbl1	32	0.391	0.015
Adrb2	54	0.308	0.026
Bst1	80	0.271	0.036
Gpx3	83	0.269	0.047
Myocd	90	0.259	0.058
Grk5	105	0.229	0.066
Ids	123	0.212	0.074
Ms4a6b	140	0.200	0.082
1810057c19rik	146	0.193	0.090
Igfbp2	149	0.190	0.097
Zfp36	218	0.159	0.100
Serpina3n	222	0.158	0.107
P2ry6	225	0.157	0.113
Adm	228	0.156	0.120
Crym	277	0.145	0.123
Ppap2a	303	0.139	0.128
Pycard	307	0.138	0.133
Kcns1	320	0.134	0.138
Cd80	321	0.134	0.144
Trim24	330	0.133	0.149
C1qtnf6	339	0.131	0.154
A330049m08rik	377	0.127	0.157
Adamts15	385	0.126	0.162
Elovl4	398	0.124	0.167
C1qa	402	0.124	0.172
Sox9	434	0.119	0.175
Htra3	455	0.116	0.179
Adam17	483	0.112	0.182
Mgll	493	0.112	0.186
Ibsp	507	0.110	0.190
C1qb	511	0.109	0.194
Bambi	516	0.109	0.199
Anxa4	551	0.105	0.201
Cd109	555	0.105	0.206
Nrk	559	0.104	0.210
Gstm1	619	0.099	0.211
Asb4	634	0.097	0.214
Pygl	654	0.095	0.217
Rasl11b	655	0.095	0.221
Cdc42ep4	674	0.093	0.224
Slc9a3r2	683	0.092	0.227
Lama1	688	0.092	0.231
Bb146404	707	0.091	0.234
Ai194308	724	0.090	0.237
Smn1	752	0.088	0.239
Alcam	772	0.087	0.242
Cst3	790	0.086	0.244
Pyp	847	0.083	0.245
2700017m01rik	870	0.082	0.247
Fgfr3	884	0.081	0.250
Mrpl34	912	0.080	0.252
C9orf46	972	0.077	0.252
Maf	981	0.077	0.255
8430420c20rik	1028	0.075	0.255
Gfm2	1030	0.075	0.259
Anxa6	1041	0.075	0.261
Isg20	1064	0.074	0.263
Auh	1068	0.074	0.266
Bsg	1100	0.072	0.267
Peg3	1179	0.070	0.266
Adam23	1208	0.069	0.268
Ezh1	1213	0.069	0.270
2810022l02rik	1214	0.069	0.273
0610011i04rik	1248	0.068	0.274
Pbx2	1257	0.067	0.277
Jup	1291	0.066	0.278
Zcwcc2	1301	0.066	0.280
Whsc2	1317	0.066	0.282
2410004l22rik	1344	0.065	0.283
Lmnb2	1388	0.064	0.284
Fndc1	1435	0.063	0.284
Rarres2	1460	0.062	0.285
Tap2	1512	0.061	0.285
Ctbs	1559	0.060	0.285
Jdp2	1574	0.059	0.287
Hck	1712	0.056	0.282
5031400m07rik	1792	0.054	0.281
Pkn1	1839	0.053	0.280
Dag1	1929	0.052	0.278
Fth1	1976	0.051	0.278
1110001e17rik	1979	0.051	0.280
Rbp4	1984	0.051	0.282
Pdcd6ip	2044	0.050	0.281
Siat7d	2050	0.050	0.283
Kcnd2	2074	0.050	0.284
2310004k06rik	2076	0.050	0.286
D19ertd678e	2106	0.049	0.286
Npdc1	2114	0.049	0.288
Fts	2116	0.049	0.290
Prickle1	2123	0.049	0.291
1110037f02rik	2171	0.048	0.291
Cdc42se1	2246	0.047	0.289
Chpt1	2261	0.047	0.290
Wwp2	2341	0.045	0.288
Dact1	2363	0.045	0.289
Rragd	2380	0.045	0.290
Irf5	2406	0.044	0.291
Nrbf2	2414	0.044	0.292
Cox4i2	2436	0.044	0.293
Bmp7	2456	0.044	0.294
1810008a18rik	2517	0.043	0.292
Asph	2533	0.043	0.293
Stat2	2550	0.042	0.294
Hoxa11	2560	0.042	0.296
Bax	2599	0.042	0.295
Sspn	2611	0.042	0.297
Ifngr2	2612	0.042	0.298
Glrx1	2672	0.041	0.297
Gba	2739	0.040	0.295
Fzd2	2759	0.040	0.296
Crtap	2772	0.040	0.297
Slc1a5	2786	0.040	0.298
Slco3a1	2831	0.039	0.297
3110040n11rik	2833	0.039	0.299
Tep1	2845	0.039	0.300
Fastk	2860	0.039	0.301
Tmed3	2869	0.038	0.302
Ephb4	2876	0.038	0.303
Asah2	2908	0.038	0.303
Pold4	2989	0.037	0.301
1110001a07rik	2995	0.037	0.302
Pcp4	3010	0.037	0.303
Mab21l2	3025	0.037	0.304

Rank = position of genes in the context of the ranked list of array genes

RMS = the ranked metric score

RES = the running enrichment score

**Table 9:** Micromass culture-derived transcripts enriched in vehicle-treated primary chondrocytes (d3 vs d15_3/4). I.

HUGO gene symbol	Rank	RMS	RES
Rabggtb	16734	-0.040	-0.271
Ube2e2	16759	-0.041	-0.270
Cd68	16769	-0.041	-0.269
H2-T23	16830	-0.041	-0.270
Derl1	16834	-0.041	-0.268
Smarcc1	16853	-0.041	-0.267
Srxn1	16856	-0.041	-0.266
Klf10	16868	-0.042	-0.264
Zfhx1b	16879	-0.042	-0.263
H2afy3	16929	-0.042	-0.264
Wisp2	16973	-0.042	-0.264
Tbl1xr1	16976	-0.042	-0.262
Ppp1r3c	16979	-0.042	-0.260
D11lgp2e	17036	-0.043	-0.261
Smpdl3b	17079	-0.043	-0.262
Dock2	17125	-0.044	-0.262
Purb	17127	-0.044	-0.260
Grn	17130	-0.044	-0.258
1110035l05rik	17139	-0.044	-0.257
Kiaa1008	17185	-0.045	-0.257
E430025l02rik	17195	-0.045	-0.256
Timm8a	17293	-0.046	-0.259
C130006e23	17307	-0.046	-0.257
Rbm10	17319	-0.046	-0.256
A230103n10rik	17347	-0.046	-0.255
Cd151	17401	-0.047	-0.256
Srf	17409	-0.047	-0.254
Cacna1s	17507	-0.048	-0.257
Ythdf1	17529	-0.048	-0.256
Ppp2r1b	17539	-0.048	-0.254
Tead2	17545	-0.048	-0.252
Igsf7	17590	-0.049	-0.252
Per3	17604	-0.049	-0.251
G1p2	17739	-0.050	-0.256
Slco2a1	17786	-0.051	-0.256
Coq7	17918	-0.053	-0.260
Rarb	17940	-0.053	-0.259
Lcp1	17954	-0.053	-0.257
Dnaja1	17987	-0.053	-0.256
Thoc3	17993	-0.054	-0.254
Cd44	18041	-0.054	-0.254
Slc41a1	18171	-0.056	-0.258
Kif11	18232	-0.057	-0.259
Hspa5bp1	18235	-0.057	-0.257
Ncf4	18290	-0.058	-0.257
Bub1b	18292	-0.058	-0.254
Cap2	18295	-0.058	-0.252
Aig1	18340	-0.059	-0.251
Rfc3	18361	-0.059	-0.250
Stmn1	18396	-0.060	-0.249
9130213b05rik	18408	-0.060	-0.247
Tyms-Ps	18432	-0.060	-0.245
Timp3	18513	-0.062	-0.247
Tiparp	18564	-0.063	-0.247
Thbs4	18627	-0.064	-0.247
Wasf1	18652	-0.064	-0.245
Nupr1	18686	-0.065	-0.244
Ezh2	18706	-0.066	-0.242
Fbxl14	18709	-0.066	-0.239
Prim1	18780	-0.067	-0.240
Insig2	18805	-0.068	-0.238
B3gnt5	18858	-0.069	-0.238
Fam60a	18963	-0.072	-0.240
H2-M3	18972	-0.073	-0.237
Gja7	18974	-0.073	-0.234
Bex2	18987	-0.073	-0.231
Tk1	19043	-0.074	-0.231
1200015n20rik	19109	-0.076	-0.231
Clecsf5	19114	-0.077	-0.228
Ms4a7	19141	-0.078	-0.226
Cdca5	19163	-0.079	-0.223
C730042f17rik	19180	-0.079	-0.220
Trim25	19194	-0.080	-0.218
Efnb2	19207	-0.081	-0.215
Apex1	19236	-0.082	-0.212
Ddah2	19243	-0.082	-0.209
Bub1	19262	-0.083	-0.206
Nup43	19263	-0.083	-0.203
Rdh10	19270	-0.083	-0.199
2610201a13rik	19330	-0.086	-0.199
Rp2h	19406	-0.089	-0.198
Tnni1	19407	-0.089	-0.195
Myog	19423	-0.091	-0.191
Osmr	19486	-0.095	-0.190
Mmp9	19524	-0.097	-0.188
Tnnt1	19525	-0.098	-0.184
Fhod3	19528	-0.098	-0.179
D930038m13rik	19537	-0.099	-0.175
Nes	19567	-0.101	-0.172
Sbk1	19571	-0.102	-0.168
Dusp9	19594	-0.103	-0.165
Akr1b8	19622	-0.106	-0.161
Pdgfrb	19663	-0.110	-0.158
Tfrc	19667	-0.111	-0.154
Moxd1	19670	-0.111	-0.149
1810008k03rik	19681	-0.112	-0.145
Cpeb1	19710	-0.115	-0.141
6720475j19rik	19716	-0.116	-0.136
Ripk4	19718	-0.116	-0.131
Itga6	19756	-0.121	-0.127
Bmp5	19775	-0.124	-0.123
Lhx9	19776	-0.124	-0.117
Pkp2	19797	-0.129	-0.113
Chrna1	19808	-0.131	-0.108
Bhlhb2	19837	-0.142	-0.103
Gp49a	19847	-0.144	-0.097
Clecsf10	19893	-0.155	-0.092
Gch1	19902	-0.159	-0.086
D0h4s114	19908	-0.161	-0.079
Cxcl1	19928	-0.170	-0.072
Ch25h	19946	-0.178	-0.065
Mkrn3	19988	-0.228	-0.057
Ptprc	20016	-0.297	-0.046
Car6	20017	-0.298	-0.032
Nr1d2	20031	-0.368	-0.017
Evi2a	20033	-0.393	0.001
Plxnc1	18075	-0.055	-0.286
Cilp2	18106	-0.055	-0.285
Brca1	18148	-0.056	-0.285
Litaf	18149	-0.056	-0.283
Bc027246	18154	-0.056	-0.281
6820424l24rik	18268	-0.057	-0.285
Hrb	18272	-0.057	-0.283
Nnat	18303	-0.058	-0.282
P2ry12	18329	-0.058	-0.282
Cdca4	18343	-0.059	-0.280
6030404e16rik	18367	-0.059	-0.279
Tfec	18429	-0.060	-0.280
Nfe2l2	18440	-0.060	-0.278
Gtf2h2	18467	-0.061	-0.277
4930469p12rik	18504	-0.062	-0.277
Cul4b	18535	-0.062	-0.276
H2afy2	18547	-0.063	-0.274
1190002n15rik	18582	-0.063	-0.274
B430218l07rik	18591	-0.063	-0.272
Rgs18	18607	-0.064	-0.270
Frk	18631	-0.064	-0.269
Slc6a9	18633	-0.064	-0.267
Tgfbr2	18687	-0.065	-0.267
Tia1	18802	-0.068	-0.270
Lgr5	18844	-0.068	-0.270
Sgpp1	18909	-0.071	-0.271
Matn2	18924	-0.071	-0.269
Sox11	18931	-0.071	-0.266
Hus1	18980	-0.073	-0.266
D930015e06rik	19028	-0.074	-0.266
Apob48r	19032	-0.074	-0.263
Av344025	19045	-0.074	-0.261
Eno2	19047	-0.074	-0.258
2610024e20rik	19053	-0.075	-0.256
Chd1l	19093	-0.076	-0.255
Emr1	19145	-0.078	-0.255
Rgs4	19200	-0.081	-0.254
D030028o16rik	19211	-0.081	-0.252
Kif2c	19216	-0.081	-0.249
Ccl3	19220	-0.081	-0.246
Trim30	19232	-0.082	-0.244
Qrsl1	19242	-0.082	-0.241
Nr3c1	19281	-0.083	-0.240
Trip13	19282	-0.084	-0.237
Dna2l	19317	-0.085	-0.236
Tcf8	19335	-0.086	-0.233
Clecsf8	19341	-0.086	-0.230
Lyzs	19422	-0.090	-0.231
Palmd	19475	-0.095	-0.230
Tjp2	19487	-0.095	-0.227
D430019h16rik	19493	-0.096	-0.224
Sesn3	19501	-0.096	-0.221
Ereg	19507	-0.096	-0.218
Cx3cl1	19523	-0.097	-0.215
Fzd6	19529	-0.098	-0.211
Sod3	19564	-0.101	-0.209
Tnnt2	19580	-0.102	-0.206
Satb1	19599	-0.104	-0.203
Cd14	19606	-0.104	-0.200
Gbp2	19607	-0.104	-0.196
Tgfbi	19609	-0.105	-0.192
Chek1	19652	-0.109	-0.190
Tm4sf1	19653	-0.109	-0.186
Igf1	19679	-0.111	-0.183
Enpp1	19695	-0.113	-0.180
Slc15a3	19704	-0.114	-0.176
Pdpn	19725	-0.117	-0.173
Dkk1	19747	-0.119	-0.169
Slk	19759	-0.121	-0.166
Ankrd1	19794	-0.128	-0.163
Trp53bp1	19801	-0.129	-0.158
C79407	19804	-0.130	-0.153
2210010l05rik	19809	-0.131	-0.149
Eps8	19815	-0.133	-0.144
Dkk2	19862	-0.147	-0.141
Arhgap18	19863	-0.147	-0.136
Twist2	19878	-0.151	-0.131
Pcdha8	19915	-0.164	-0.126
Il4r	19926	-0.169	-0.121
Mdm1	19931	-0.172	-0.115
Phlda1	19957	-0.188	-0.109
Bhlhb5	19960	-0.192	-0.102
C130076o07rik	19964	-0.196	-0.095
5830411e10rik	19974	-0.207	-0.088
Ptpre	19989	-0.228	-0.080
Trib3	19990	-0.235	-0.071
9230117n10rik	19994	-0.241	-0.062
Pcdhb7	19998	-0.249	-0.053
Mmp3	20001	-0.252	-0.044
Cd34	20009	-0.274	-0.034
Thbd	20022	-0.310	-0.023
A830016g23rik	20023	-0.323	-0.011
Ahr	20028	-0.336	0.001

Rank = position of genes in the context of the ranked list of array genes

RMS = the ranked metric score

RES = the running enrichment score

Four different patterns were observed when comparing DEX treatment and micromass differentiation data sets for gene enrichments scores (Figure 4B). First, similar gene sets were indeed enriched in both day 15 micromass and DEX-treated monolayer cultures, and core genes contributing to the normalized enrichment scores were similarly overlapping between the two data sets in results with low FDR. For example, ECM genes were enriched with both DEX treatment and the day 15 micromass phenotype. Other gene sets following this enrichment pattern included genes involved in integrin function, angiogenesis, catalytic activity, IGF related, adipocyte and cartilage, all of which have a precedent for being involved in chondrocyte maturation [28,49,80,81]. The enrichment of angiogenic transcripts with DEX treatment was unexpected since DEX was shown to have anti-angiogenic roles in cartilage; however, upon closer examination of the genes contributing to the enrichment score, *Vegf*, which is thought to be a central angiogenic factor in endochondral ossification [82], was excluded from the core enrichment genes and had the lowest correlation with the DEX phenotype in that gene set. In contrast, *Vegf *was enriched in the growth factor data set which positively correlated with the vehicle control and not DEX treatment (Table 7).

Gene sets associated with the actin cytoskeleton, tumour suppressors, structure, cytoplasmic genes, hepatocyte markers and dual specificity phosphatases (DUSPs) were enriched in the DEX data set and the phenotype positively correlated with day 3 of micromass culture. The identification of DUSPs was particularly interesting since DEX has been shown to induce genes encoding for these proteins [77,83,84]. DUSPS counteract the activation of MAP kinase pathways, known regulators of chondrocyte differentiation [85], and are thought to mediate DEX's anti-inflammatory functions and to influence hepatic gluconeogenesis [83,86,87].

Additional comparisons identified genes that show enrichment in day 15 micromass cultures and downregulation with DEX treatment. These include the previously identified chemokines, cytokines and interleukins. A final trend in similarly enriched gene sets identified lists that were negatively correlated both with the DEX phenotype and day 15 of micromass cultures. Only transcriptional repressors and molecules involved in the extracellular signal-regulated kinase (ERK) pathway were identified. This pattern is consistent with DEX's anti-proliferative functions, as another study showed that DEX decreases ERK phosphorylation and thus cell cycle progression in a pre-osteoblast cell line [77]. Altogether this analysis shows that DEX regulation of growth plate chondrocyte differentiation is multifaceted. The patterns identified here are in agreement with a dual role of DEX in maintenance of the cartilage phenotype and delay in the cartilage-to-bone transition, as we suggested above.

We also wanted to determine whether DEX target genes identified in the current study were similar to DEX-responsive genes identified in alternate studies, in different cell types [88]. Out of a total of twelve microarray studies evaluating the transcriptional effects of DEX treatment on a specific tissue or cell type, only ten genes were common to at least three of the chosen DEX studies. Specifically, bone morphogenetic protein 2 (*Bmp2*), delta sleep inducing factor 1 (*Dsip1*), beta-2 microglobulin (*B2m*), neuroepithelial cell transforming gene 1 (*Net1*), TNFAIP3 interacting protein 1 (*Tnip1*), bone marrow stromal cell antigen 2 (*Bst2*), B-cell leukemia/lymphoma 6 (*Bcl6*), nuclear factor of kappa light chain gene enhancer in B-cells inhibitor, alpha (*Nfkbia*), FK506 binding protein 5 (*Fkbp5*) and B-cell translocation gene 1, anti-proliferative (*Btg1*) were identified. It therefore appears that while DEX affects similar functional categories across various species, tissue types and experimental conditions, the individual genes that respond to DEX treatment are variable. These results also reinforce the paradigm that GC regulation is inextricably linked to its physiological context [88-99].

### Analyses of GC response elements in dexamethasone target genes in chondrocytes

Classical genomic GC action is thought to be mediated by a cytoplasmic GR that modifies transcription upon binding its cognate ligand and translocating to the nucleus. In the nucleus, the GR binds a GRE sequence. GR can both activate and repress transcription, depending on the GRE variant present in the regulatory regions of GC target genes [100]. Binding to composite GREs involves homodimerization of the GR to bind a non-palindromic consensus sequence comprised of two GR binding sites and is generally associated with transcriptional activation. In some instances, however, GR can function to block access or activity of transcription factors within promoter regions of certain genes, thus impeding transcription [101]. GR are also able to bind a modified GRE consisting of composite GRE half-sites, termed negative GREs, since they have documented roles in transcriptional repression. Variations on the genomic functions of GC include transcriptional regulation at the level of protein-protein interactions between the GR and other transcription factors, co-activators or co-repressors. In addition to the GRE-dependent roles, the GR is capable of interacting with other co-activators and repressors to influence transcription indirectly [102,103].

**Figure 5:**
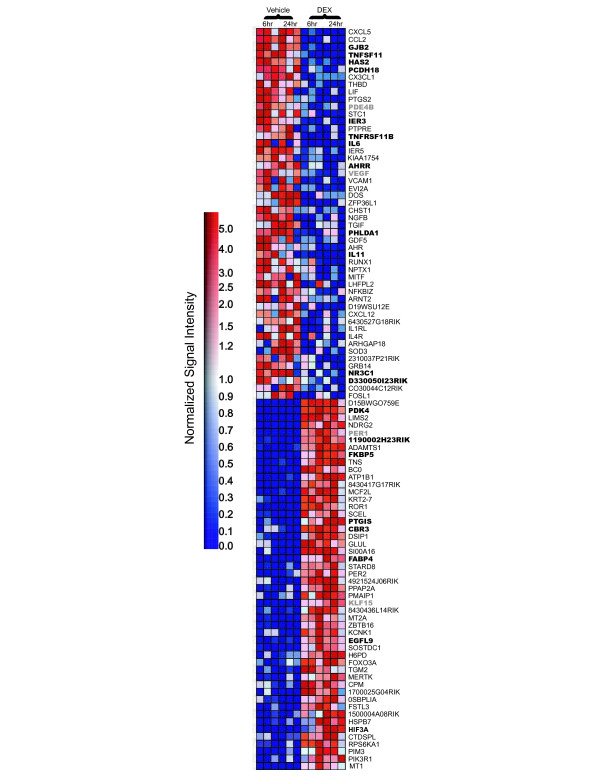
**Heat map of top 100 probe sets determined by GSEA analysis**. GSEA-derived heat maps of the top 100 differentially expressed probe sets enriched with DEX or the vehicle control are shown (B). Expression profiles for all experimental replicates are shown for each time point. Genes containing a putative GRE are shown in bold, and examples of genes that do not contain GREs but have been documented as targets of DEX regulation are depicted by bold gray lettering. Signal intensities are illustrated by varying shades of red (up-regulation) and blue (down-regulation).

The 100 most highly expressed probe sets with greatest enrichment in the DEX or vehicle-treated chondrocytes are shown in Figure 5. Probe sets identified in this analysis included both known cartilage markers and established DEX target genes such as *Vegf, Ibsp*, *Bglap2 *and *Fkbp5 *[49,63-66,104,105]. We examined the proximal promoter regions of three separate gene lists, the top 100 DEX-responsive transcripts generated by GSEA analysis (Figure 5), the 22 091 probe sets deemed expressed in primary chondrocyte cultures and the 1158 transcripts deemed differentially expressed between DEX and vehicle treated cultures by one-Way ANOVA. Specifically, we searched the 9990 base pairs upstream regulatory regions in this list for the composite GRE consensus sequence. We identified putative GRE sequences in many genes, including *Fkbp5*, pyruvate dehydrogenasekinase (*Pdk4*), RANKL (*Tnfsf11*), Interleukin 6 (*Il6*) and prostaglandin I2 synthase (*Ptgis*) (bold in Figure 5). However, the majority of DEX-regulated probe sets such as prostaglandin-endoperoxide synthase 2 (*Ptgs2*), phosphodiesterase 4A (*Pde4*), *Vegf*, Period homolog 1 (*Per1*) and Krüppel like factor 15 (*Klf15*) do not appear to contain a GRE in the first 10 kilobases and may by regulated by DEX via a GRE-independent mechanism, through a GRE that deviates from the consensus GRE sequence or through GREs at other locations in the gene.

Examination of all lists generated similar results in that approximately 16–20% of all probes contained the consensus GRE. Consequently, we cannot exclude the presence of less conventional GRE loci in the transcripts, or the presence of GREs that deviate from the consensus sequence or are located outside the queried sequence. Since many of the genes affected at the 6 hr time point encode transcription factors, it is likely that a large proportion of the genes that only change after 24 hrs are regulated indirectly by DEX, through altered expression of these transcription factors and other regulatory proteins (e.g. phosphatases and cytokines, as discussed above).

Functional analysis is required to unequivocally evaluate the contribution of GRE-dependent mechanisms to GC regulation in chondrocytes. In addition to the genomic functions of GC, non-genomic modes of GC regulation have been documented. Non-genomic mechanisms are thought to occur through specific and non-specific mechanisms. Specific non-genomic GC regulation occurs through the classical GR and its cytoplasmic heteroprotein complex or non-classical GRs such as membrane GR [106-109]. Conversely, non-specific non-genomic mechanisms rely on the physiochemical properties of GC and the phospholipid bilayer (Buttgereit and Scheffold, 2002). Further, studies in which candidate molecules are selected and characterized in depth are imperative to discern the specific regulatory mechanisms occurring in chondrocytes.

## Conclusion

This study elucidates the downstream transcriptional impact of pharmacological GC exposure on developing chondrocytes. We have identified a small subset of transcripts containing putative GREs in cartilage, but it appears that GRE-independent or indirect mechanisms of GC regulation also contribute to GC regulation in primary chondrocyte monolayer cultures. In addition, traditional microarray analysis methods and gene class testing point to a dual role for pharmacological GC doses in chondrocytes. DEX acts in a gene class-specific manner in cartilage in which it promotes the expression of ECM and metabolic transcripts necessary for maintaining the chondrocyte phenotype while simultaneously downregulating cytokines and growth factors which stimulate the cartilage to bone transition. Understanding the implications of gene expression changes and integrating them into the network of molecules controlling cartilage development continues to be challenging, but robust analytical methods will prove to be useful in constructing the networks of gene interactions and understanding the complex nature of GC signaling in the skeleton. The ultimate objective of this study will be to translate these findings into more efficacious therapeutic GCs.

## Methods

### Animals and Materials

Timed-pregnant CD1 mice were purchased from Charles River Laboratories at embryonic day E15.5 mice (E15.5). Dexamethasone was obtained from Calbiochem and reconstituted in Dimethyl sulfoxide (DMSO, vehicle) according to the manufacturer's instructions. Cell culture materials and general chemicals were obtained from Invitrogen, Sigma or VWR unless otherwise stated.

#### Primary cell culture and dexamethasone-treatment

Tibiae, femurs and humeri were isolated from E15.5 mouse embryos and placed in α-MEM media (Invitrogen) containing 0.2% Bovine Serum Albumin (BSA), 1 mM β-glycerophosphate, 0.05 mg/ml ascorbic acid and penicillin/streptomycin and incubated at 37°C in a humidified 5% CO_2 _incubator overnight. The following morning media was removed and the bones placed in 4 ml of 0.25% trypsin-EDTA (Invitrogen) for 15 min at 37°C. Trypsin was subsequently replaced with 1 mg/ml collagenase P (Roche) in DMEM/10% fetal bovine serum (Invitrogen), and cells were incubated at 37°C with rotation at 100 rpm for 90 min. Following digestion, the cell suspension was centrifuged for 5 min at 1000 rpm, and the collagenase containing supernatant was decanted. Chondrocytes were resuspended in media containing 2:3 DMEM:F12, 10% fetal bovine serum, 0.5 mM L-glutamine, and penicillin/streptomycin (25 units/ml). Cells were seeded in 6-well NUNC plates at a density of 2.5 × 10^4 ^cells per ml and incubated overnight. Primary monolayer chondrocytes were treated with 10^-7 ^M dexamethasone (DEX) or the DMSO control (vehicle) diluted in fresh media supplemented with 0.25 mM ascorbic acid (Sigma) and 1 mM β-glycerophosphate (Sigma) and incubated for up to 24 hrs. Micromass cultures were completed as previously described [50].

#### Cell counting studies

Chondrocytes were isolated and seeded in 24-well NUNC plates (Nunc Inc.) at a density of 16 000 cells/cm^2^. Cells were cultured, treated and enzymatically digested as described with some modifications. Collagenase digestion occurred for 5 minutes followed by mechanical digestion to liberate cells from the ECM. Cells were counted with a hemocytometer in triplicate with a minimum of 3 individual wells per treatment and three independent cell isolations.

#### RNA isolations and quantitative real-time PCR

All RNA protocols were completed as previously outlined [50]. Total RNA was isolated at 6 hrs and 24 hrs after treatment using the RNeasy mini extraction kit (Qiagen) according to the manufacturer's instructions. RNA quantity and integrity was assessed using the Bioanalyzer 2000 system (Agilent). Quantitative real-time polymerase chain reaction (qRT-PCR) amplification was completed using the ABI Prism 7900 Sequence Detection System (Applied Biosystems). Triplicate reactions were executed for each sample of each of three independent trials. The TaqMan one-step master mix kit (Applied Biosystems) with gene-specific target primers and probes were used for amplification. The collagen X (*Col10a1*) probe and primer set (forward primer 5'-ACGCCTACGATGTACACGTATGA-3', reverse primer 5'-ACTCCCTGAAGCCTGATCCA-3', 6-FAM-5'-AGTACAGCAAAGGCTAC-MGBNFQ) was designed with PrimerDesigner 2.0 software (Applied Biosystems) [79]. TaqMan GAPDH control reagents for house-keeping gene glyceraldehyde-3-phosphate dehydrogenase (*Gapdh*, forward primer 5'-GAAGGTGAAGGTCGGAGTC; reverse primer 5'-GAAGATGGTGATGGGATTTC; probe JOE-CAAGCTTCCCGTTCTCAGCC-TAMRA) was used as an internal amplification control. Probes for Indian hedgehog (*Ihh*), Tissue inhibitor of matrix metalloproteinase 4 (*Timp4*), Cyclin-dependent kinase inhibitor 1C (*Cdkn1c*, p57), Integrin beta like 1 protein (*Itgbl1*), GC receptor (*Nr3c1*), Integrin beta 1 (*Itgb1*) and Kruppel-like factor 15 (*Klf15*) were assayed using the TaqMan^® ^gene expression assays in accordance with the manufacturers directions. Amplified transcripts were quantified using the standard curve method, and the relative transcript abundance was determined by calculating the quotient of the gene of interest and equivalent *Gapdh *values.

### Microarray analysis

Total RNA was extracted from control and DEX-treated cultures at 6 hr and 24 hr following treatment, in three independent experiments. RNA integrity and quantity was assessed using the Agilent 2000 Bioanalyzer system, and RNA samples were subsequently hybridized to the MOE 430 2.0 mouse chip from Affymetrix^© ^containing 45 101 probe sets as described [50]. Bioanalysis, microarray hybridization, scanning and preliminary MAS 5.0 normalizations were completed at the London Regional Genomics Facility. Data were deposited in the GEO database (NCBI; accession number GSE7683).

### Data normalization

Microarray data were pre-processed using the GC-RMA algorithm in Genespring GX*. Expression values were further filtered by retaining only those probe sets with expression values of at least 50 in at least 25% of all conditions, thus generating a list of 22 091 probe sets. To assess differential gene expression between treatments at both the 6 and 24 hr time points, a Welch ANOVA test with a p-value cut-off of 0.01 and a 5% false discovery rate (FDR) reduced the data to 1158 probe sets. Subsequent 1.5-, 5- and 10-fold change filters produced lists of 162, 21 and 7 probe sets for the 6 hr time point and 399, 53 and 19 probe sets for the 24 hr time point, respectively.

The same data set was normalized in parallel using Robust Multichip Analysis using RMAEXPRESS software v.0.4.1 developed by B. Bolstad, University of California, Berkeley [110]. Background adjustment and quantile normalization parameters were selected for data processing. Logarithmically transformed expression values were used to implement Gene Set Enrichment Analysis (GSEA).

#### Gene set enrichment analysis (GSEA)

The GSEA algorithm was implemented with GSEA v2.0 software [51,52]. Ranked expression lists were derived from RMAEXPRESS and GeneSpring GX^® ^7.3.1. Briefly, the GSEA algorithm ranks all array genes according to their expression under each experimental condition. The resulting ranked metric score (RMS) is therefore a function of the correlation between a gene's signal intensity, the experimental conditions in question and all other genes in the data set. An enrichment score (ES) is then calculated for an a priori gene list or gene set that is associated with a particular molecular classification. In our analysis, gene sets were created from different functional groupings, molecular classifications, tissues, and other microarray screens. A Ranked enrichment score (RES) which determines the extent to which a given gene from a gene set is represented at the extremes of the ranked gene list is then calculated. Specifically, this value is obtained by walking along the ranked list using a cumulative sum statistic which increases when a member of a particular gene set is found in the ranked gene list and is coordinately penalized when it does not appear in the gene set. A null distribution of ES is subsequently generated by permutation filtering to evaluate the statistical significance of the observed RES values. Permutation filtering randomly assigns the experimental conditions or class labels (i.e., DEX versus vehicle) to the different microarray samples. After this procedure has been repeated for each gene set, the ES are normalized (NES) to account for differences in gene set size. The false discovery rate (FDR) is then calculated relative to the NES values to determine the false-positive rate. Significant FDR and p-values were less than 25% and 0.001, respectively in accordance with GSEA recommendations.

#### Gene set creation

Gene sets were generated using the probe set search tool and the molecular function class of Gene Ontology annotations in GeneSpring GX. Additional gene sets were created using lists from pairwise comparisons between day 3 and 15 of a previously generated micromass data set (James et al., 2005), and publications that identified DEX target genes in other cartilage array screens, other tissue types and experimental systems. A total of 2119 probe sets showing a minimum 1.5-fold change in gene expression were used in the analysis. Probe set redundancy was eliminated in all gene sets using the CollapseDataset function in the GSEA program. All probe set identifiers were assimilated to the Human Genome Organization (HUGO) annotations. Probe sets lacking corresponding HUGO annotations were excluded from the analysis. Default parameters were used to execute the analysis and median values taken to represent the range of duplicated probe sets for a given gene. A total of 77 user-defined gene sets were generated from GeneSpring derived Gene Ontology annotations for various molecular classifications and probe sets of differentially expressed genes between days 3 and 15 of micromass culture (James et al., 2005).

#### Glucocorticoid response element (GRE) analysis

Putative GRE were identified with the GenespringGX mouse genome9999 application which allows sequences up to 9999 bp upstream of the transcriptional start sites of all annotated MOE4302.0 transcripts to be interrogated for transcription factor binding sites. The GR consensus sequence GGTACAnnntgttCT [111] was queried from 10 bp to 10 000 bp upstream of the transcriptional start sites of available probe sets. The GRE consensus sequence was screened against 10 748 probe sets derived from the list of 22 091 reliably expressed probe sets exhibiting homology to upstream regulatory regions annotated in the program. Only exact matches were retained for subsequent analyses out a total of 1,073,741,824 tests.

## Abbreviations

DEX: Dexamethasone; GSEA: gene set enrichment analysis; RES: ranked enrichment score; RMS: ranked metric score, ES: enrichment scores; NES: normalized enrichment score, SOM: self-organizing maps; FDR: false discovery rate; GR: glucocorticoid receptor

## Competing interests

The author(s) declare that they have no competing interests.

## Authors' contributions

CGJ completed cell culture experiments, data analysis, real-time PCR and drafted the manuscript. VU completed cell culture experiments. JT and TMU contributed to the design of the study and the writing of the manuscript. FB conceived of the study and contributed to the writing of the manuscript. All authors read and approved the final manuscript.
